# Nanobodies in biomedicine: from molecular characteristics to fabrication and clinical translation

**DOI:** 10.1016/j.mmr.2026.100009

**Published:** 2026-04-15

**Authors:** Cheng-Kun Cao, Xin-Yi Xu, Fei Liang, Min Yao, Yuan-Yuan Chen, Xiao-Kun Li, Zhi-Jian Su

**Affiliations:** a School of Pharmaceutical Sciences, Wenzhou Medical University, Wenzhou 325000, Zhejiang, China; b National Engineering Research Center for Cell Growth Factor Drugs and Protein Biologics, Wenzhou 325000, Zhejiang, China; c National Key Laboratory of Macromolecular Drug Development and Manufacturing, Wenzhou 325000, Zhejiang, China

**Keywords:** Nanobody (Nb), Molecular structure, Functional characteristics, Preparation methods, Diagnostic and therapeutic applications

## Abstract

Nanobodies (Nbs), the antigen-binding single-domain fragments derived from camelid heavy-chain antibodies (Abs), have rapidly become a focus of biomedical research due to their compact size, high stability, strong antigen affinity, and ease of molecular engineering. This review systematically outlines their structural and functional features, current strategies for acquisition, screening, optimization, and large-scale production, and comprehensively discusses their wide-ranging applications in therapeutics, diagnostics, and basic research. Specifically, Nbs have shown outstanding efficacy in tumor, toxin, infectious, and cardiovascular disease treatments, while serving as versatile tools for molecular imaging, biosensing, protein purification, structural analysis, and intracellular regulation. The challenges of immunogenicity, off-target effects, and industrial-scale manufacturing are also critically examined. Furthermore, the integration of artificial intelligence in structure prediction, de novo design, and immunogenicity assessment has opened powerful new avenues for rational Nb engineering. Combined with emerging technologies such as gene therapy, nanomaterial delivery, and multispecific architectures, these advances promise to accelerate clinical translation. Overall, Nb technology is poised to become a cornerstone of next-generation precision medicine and biotechnology, offering innovative solutions for disease diagnosis, targeted therapy, and molecular discovery.

## 1 Background

Heavy-chain antibodies (Abs), identified in camelids and sharks, are a distinct type of immunoglobulin (Ig) [1]. Nanobodies (Nbs) are antigen-binding single-domain fragments derived from naturally occurring heavy-chain Abs in camelids [2]. Since their discovery in the early 1990s, Nbs have gained considerable attention due to their small molecular weight, exceptional stability, high antigen affinity, and ease of genetic manipulation. These unique characteristics distinguish them from traditional monoclonal Abs and confer broad potential applications in diverse biomedical fields [3], [4]. In sharks, the similar antigen-binding single-domain fragments are known as variable new antigen receptors (VNARs) [5]. However, camelid-derived Nbs have attracted broader biomedical interest because they are easier to obtain and manipulate, exhibit favorable biophysical characteristics, and generally display lower immunogenicity compared to VNARs [6].

Over the past three decades, extensive research has been devoted to elucidating the structural foundations and functional characteristics of Nbs. Meanwhile, diverse strategies for Nb fabrication have been continuously developed and refined. These advances have greatly facilitated the broad exploration of Nbs in basic scientific research, diagnostics, and therapeutics [7], [8], [9]. Currently, the rapid emergence of artificial intelligence (AI)-driven protein design and structure prediction tools has opened new avenues for accelerating Nb discovery, optimizing binding properties, and engineering multifunctional Nb constructs [10], [11], [12].

The present review aimed to systematically examine the molecular structures and functional characteristics of Nbs, outline the major current preparation strategies, and summarize their diverse applications across the biomedical field. Furthermore, by integrating recent research progress with AI-driven innovations in protein engineering, we propose potential future directions for Nb development in precision medicine and biotechnology, with the goal of providing valuable reference and inspiration for related research endeavors.

## 2 Evolution of antibodies therapeutics and the rise of nanobodies

**Fig. 1:**
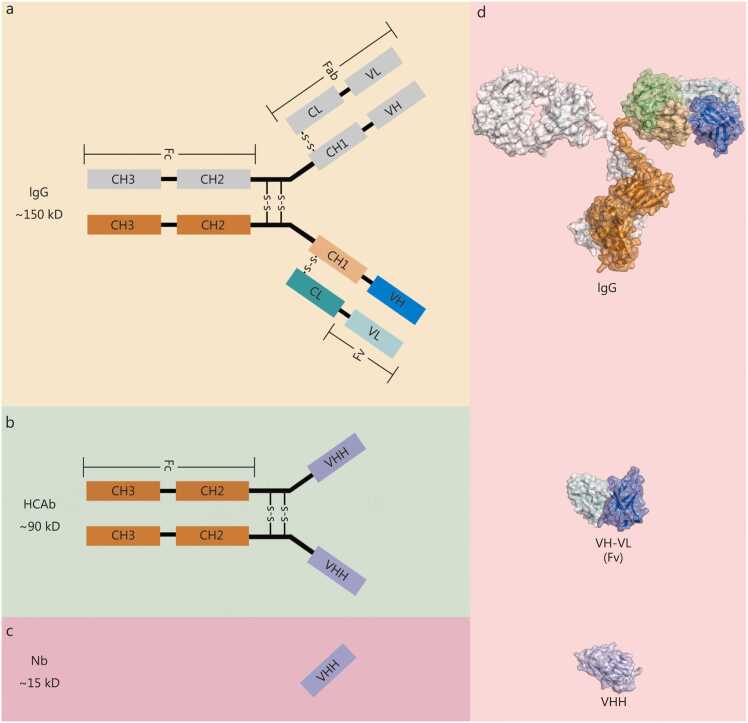
Structural and size comparison of antibodies (Abs). **a** Diagram of immunoglobulin G (IgG) structure, depicting its Y-shaped configuration with heavy and light chains. **b** Diagram of heavy-chain antibody structure, composed exclusively of heavy chains. **c** Diagram of a nanobody (Nb) structure, illustrating a single variable domain of heavy chain of heavy chain antibody (VHH). **d** Size comparison of IgG, the variable fragment (Fv) formed by VH-VL of IgG, and VHH at the same scale. The protein structural data for IgG (PDB ID: 1IGT) and VHH (PDB ID: 8FTG) used in panel d of this figure are referenced from previous publications and visualized with PyMOL (v3.0.6) [21], [22]. VH is marked in blue; VL is marked in light blue; CHs are marked in light orange and orange colors; CL is marked in green; VHH is marked in purple, and the half of IgG is indicated by gray shading. CH. Constant region of heavy chain; CL. Constant region of light chain; VH. Variable region of heavy chain; VL. Variable region of light chain; Fab. Fragment antigen-binding; Fc. Fragment crystallizable.

As a crucial component of the biopharmaceutical industry, Ab-based products have demonstrated remarkable value in various clinical diagnostic and therapeutic applications owing to their high specificity and affinity [13], [14]. The use of exogenous Abs in humans has a long history. As early as the 19th century, Behring and colleagues elicited immune responses by injecting bacterial toxins into animals, thereby obtaining immune sera that were successfully applied to the treatment of diphtheria and tetanus, saving countless lives [15]. This milestone marked the initial introduction of polyclonal Abs into scientific research and clinical practice. In the mid-20th century, advances in proteolytic cleavage and chemical analysis techniques enabled scientists to elucidate the basic molecular architecture of Abs, comprising two heavy chains (H chains) and two light chains (L chains) linked by disulfide bonds to form a “Y”-shaped tetrameric structure **(**Fig. 1**a)**. This discovery established the molecular foundation for subsequent in-depth research in immunology and molecular biology [16]. Subsequently, the proposal of the “immune network theory” further advanced understanding of Ab diversity mechanisms [17], while the development of hybridoma technology enabled the *in vitro* production of monoclonal Abs (mAbs) [18]. These breakthroughs revolutionized Ab research and application, leading to an era of bioengineering-driven innovation characterized by targeted design and large-scale production. Since the approval of the first therapeutic Ab drug, Orthoclone OKT3, for clinical use in 1986 [19], the field of Ab drug research and development has entered a period of rapid and sustained advancement. To date, more than 200 Ab-based therapies have been approved for the treatment of human diseases [20], making them a core component of the modern biopharmaceutical industry.

According to the functional specialization of distinct domains within the Ab molecule, Abs can be divided into the fragment antigen-binding (Fab), which specifically recognizes and binds target antigens, and the fragment crystallizable (Fc), which mediates interactions with immune effector components to promote antigen clearance [23]. Structurally, each molecule comprises two heavy chains and two light chains linked via disulfide bonds. The heavy chain is further divided into a constant region (CH) and a variable region (VH) [24]. Based on differences in heavy-chain structure, mammalian Abs can be classified into 5 major subtypes: IgG, IgM, IgA, IgE, and IgD. These isotypes differ in the amino acid sequences and number of the CH domains, the presence and structure of the hinge region, Ab valency, and other physicochemical characteristics [25]. Among these, IgG is the predominant subtype generated during adaptive immune responses and is also the most abundant Ab in serum, accounting for approximately 75% of the total Ab [26]. The light chain consists of a continuous structure comprising one constant region (CL) and one variable region (VL) [27]. Together, the variable regions of both heavy (VH) and light (VL) chains form the antigen-binding site, conferring specificity for diverse epitopes.

In 1993, scientists first discovered a unique class of naturally occurring heavy-chain Abs in camelids. These Abs consist solely of heavy-chain dimers and completely lack light chains [28] **(**Fig. 1**b)**. Moreover, their heavy chains are devoid of the CH1 domain that, in conventional IgG Abs, mediates pairing with the light chain. Instead, the corresponding position is occupied by a single variable antigen-binding domain, designated as the variable domain of heavy chain of heavy-chain Ab (VHH) [29]. The crystal structure of VHH is ellipsoidal, with a diameter of approximately 2.5 nm and a length of about 4.2 nm [30]. Its molecular mass ranges from 12–15 kD, roughly one-tenth that of a full-size IgG molecule [21], [22], [31] **(**Fig. 1**c, d)**. Structurally and functionally, VHH is similar to the Fv region of conventional Abs, and equivalent antigen-binding specificity and affinity [32]. Based on these characteristics, VHHs are commonly referred to as Nbs or single-domain Abs (sdAbs).

Since the approval of the first Nb-based therapeutic, Caplacizumab, by the FDA in 2019 [33], Nbs have rapidly gained prominence as an innovative molecular platform with exceptional potential in precision medicine and biotechnology. Their unique physicochemical properties and high versatility are expected to position Nbs as powerful successors to conventional Ab therapeutics, with remarkable prospects for future advancements in both scientific research and clinical applications.

## 3 Molecular structure and functional characteristics of nanobodies

### 3.1 Structure of nanobodies

**Fig. 2:**
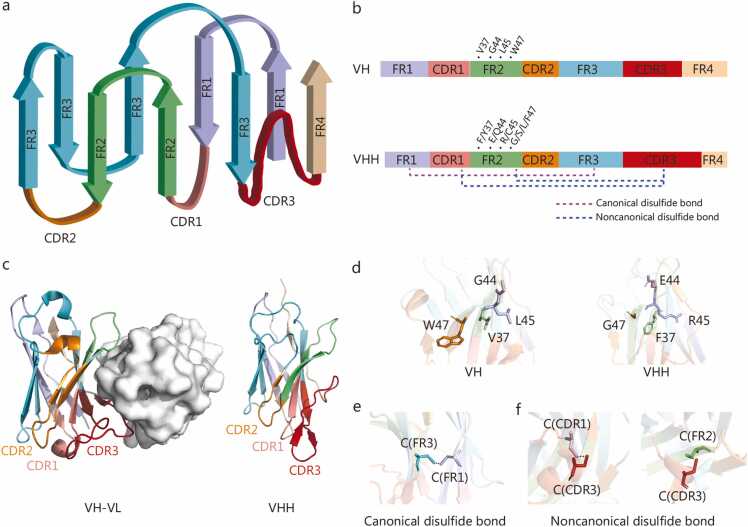
Nanobody (Nb) structure and comparative analysis. **a** Structural diagram of a Nb (VHH). **b** Schematic of FRs and CDRs in a Nb. **c** Crystal structure comparison of the VH-VL domains of conventional IgG and VHH. **d** Comparison of specific amino acid residues in FR2 between VH and VHH. **e** Schematic of the canonical disulfide bond in VHH. **f** Schematic of the noncanonical disulfide bond in VHH. The protein structural data for VH-VL (PDB ID: 6WPS), VHH illustrating the canonical structure of a VHH and the canonical disulfide bond (PDB ID: 7KSG) and VHH illustrating the noncanonical disulfide bond (PDB ID: 6DBA and 5IVO) used in panels c, d, e, and f of this figure are referenced from previous publications and visualized with PyMOL (v3.0.6) [36], [37], [38], [39]. FR1 is marked in purple; FR2 is marked in green; FR3 is marked in blue; FR4 is marked in light apricot; CDR1 is marked in pink; CDR2 is marked in orange, and CDR3 is marked in red. FR. Framework region; IgG. Immunoglobulin G; VH. Variable region of heavy chain; VL. Variable region of light chain; VHH. Variable domain of heavy chain of heavy chain antibody; CDR. Complementarity-determining region.

Nbs represent the variable domains of heavy-chain Abs, and their three-dimensional structures are highly analogous to the variable domains of conventional Abs. For instance, both molecules consist of tightly packed parallel β-sheet layers [34] **(**Fig. 2**a)** and contain four highly conserved framework regions (FR1-FR4) as well as three hypervariable complementarity-determining regions (CDR1-CDR3), which together determine antigen specificity [35] **(**Fig. 2**b)**. Despite these overall structural similarities, several significant differences distinguish the structure of Nbs from the VH domain of conventional Abs.

#### 3.1.1 Structural features of framework regions (FRs) in nanobodies

Although the FRs of both VH and VHH act as structural scaffolds, the VH requires association with a VL chain to stabilize the Fab fragment, whereas the VHH domain exhibits greater structural flexibility and can independently support CDR diversity and antigen binding [36], [37], [40] **(**Fig. 2**c)**. Notably, the FR2 segment of VH typically contains four highly conserved hydrophobic residues (V37, G44, L45, and W47), while in VHH these positions are substituted with hydrophilic residues such as F37/Y37, E44/Q44, R45/C45, and G47/S47/L47/F47. These substitutions enhance Nb solubility and compensate for the absence of a light chain, collectively contributing to improved molecular stability [36], [37], [41], [42], [43] **(**Fig. 2**d)**. In conventional Abs, FR2 is usually buried within the molecule and does not directly participate in antigen binding. However, in certain VHH molecules, when CDR3 forms a β‑hairpin structure, FR2 can directly engage antigen residues and play an important role in epitope recognition [44], [45]. Moreover, cysteine residues located between FR1 and FR3 can form a canonical inter‑β‑sheet disulfide bond that enhances structural stability [37], [46] **(**Fig. 2**e)**. Some VHH variants also contain additional cysteine residues in FR2 that can form a noncanonical disulfide bond with cysteines in CDR3, thereby further stabilizing the Nb structure [38], [39], [47] **(**Fig. 2**f)**.

#### 3.1.2 Structural features of complementarity-determining regions (CDRs) in nanobodies

The CDRs represent the principal structural elements responsible for antigen recognition and binding. The amino acid composition and sequence of these regions determine the specificity of Abs [48]. Among the three CDRs in VHH, CDR1 and CDR2 are relatively short, whereas CDR3 exhibits markedly increased length and sequence diversity [49] **(**Fig. 2**b)**. Typically, VHH CDR3s consist of approximately 15 residues, with the longest sequences approaching 30 amino acids, substantially longer than the average 12‑residue CDR3 in conventional VH domains [50]. Structurally, the CDR3 of VHH usually folds toward FR2, corresponding to the side of FR2 in VH that is used for binding to the VL [36], [37], [51] **(**Fig. 2**c)**. The CDR3 also promotes global structural stability by forming a hydrophobic core with adjacent FR residues [52]. Furthermore, the CDR1 of VHH can form non‑canonical disulfide linkages with CDR3, thereby reinforcing overall molecular rigidity [38], [39], [53], [54] **(**Fig. 2**f)**. Functionally, among the amino acid residues involved in Ab binding, the proportion of CDR3 residues is significantly higher in VHH (approximately 46%) than in VH-VL (approximately 38%), and their types are also more diverse [55]. In conventional Abs, six CDRs from the VH and VL domains jointly form the antigen‑binding site, which usually presents a relatively flat binding surface [56]. By contrast, the extended CDR3 loop in VHH often generates a protruding or convex paratope, enabling the Nbs to access recessed or hidden epitopes within target antigens [57]. For example, a study on anti‑lysozyme Nbs has demonstrated their preferential binding to concave regions of the lysozyme molecule rather than to planar surface sites [58].

### 3.2 Characteristics of nanobodies

Nbs possess a set of distinctive biophysical and biochemical features that underpin their expanding applications in diagnostics, therapeutics, and biotechnology. These features include high antigen-binding affinity and specificity, exceptional physicochemical stability and solubility, intrinsically low immunogenicity, and favorable pharmacokinetic and engineering properties derived from their small molecular size.

#### 3.2.1 Antigen-binding affinity and specificity

Despite the absence of the light chain-associated epitopes found in conventional Abs, Nbs maintain extremely strong binding affinity through conformational flexibility and the extension or twisting of their CDR3 loops [59], [60], [61]. It was reported that high-affinity Nbs derived from immune libraries can display equilibrium dissociation constants (KD) in the range of 50–100 pmol/L, and in some cases as low as 12 pmol/L [62]. Furthermore, the Nbs targeting green fluorescent protein (GFP) fail to recognize cyan fluorescent protein (CFP), a structurally and sequentially similar homolog, unless residue I146 in CFP is mutated to N146, underscoring the exceptional target specificity of Nb-antigen interactions [63], [64].

#### 3.2.2 Physicochemical stability

Nbs demonstrate outstanding thermal and chemical stability, far exceeding that of conventional Abs. Certain Nbs can refold and fully regain functionality after denaturation in 7 mol/L guanidine hydrochloride or 10 mol/L urea [65]. This intrinsic robustness is largely attributed to internal disulfide linkages that stabilize the molecular conformation under harsh conditions. Moreover, Nbs tolerate high temperatures (60–80 °C) with minimal loss of activity [66]. This property has been exploited for simplified purification by heat treatment, yielding results comparable to affinity chromatography [67]. Furthermore, engineered Nbs with additional disulfide bonds also exhibit heightened structural rigidity and enhanced resistance to proteolytic digestion, such as by pepsin [68].

#### 3.2.3 High solubility

Conventional Abs are soluble glycoproteins whose FRs contain numerous hydrophobic residues required for heavy and light chain association, often predisposing the molecules to aggregation when environmental conditions fluctuate [69]. In contrast, Nbs replace these hydrophobic interface residues with hydrophilic amino acids, markedly improving solubility. This adaptation compensates for the absence of a light chain and further enhances molecular stability and folding robustness [43]. A representative example is that by introducing as few as two hydrophilic residues (G44E and L45R) from the FR2 region of camels into human VH (a process referred to as camelization), the solubility of the latter can be significantly enhanced without affecting its targeting affinity [70]. Similarly, the sdAbs obtained from immunized camels have hydrophobic residues in their FR regions that are similar to those of human VH. By introducing VHH-like mutations (V37Y, G44E, L45R, and W47L), sdAbs with high solubility while maintaining their original functions were obtained [71].

#### 3.2.4 Low immunogenicity

Traditional Abs contain an Fc region that binds to Fcγ receptors (FcγR) and activates the classical complement pathway, mediating immune effector functions while also contributing to inflammation and immunotoxicity [72], [73]. Nbs, by lacking an Fc domain, circumvent these Fc-mediated immune responses and consequently exhibit greatly reduced risk of immune-related adverse effects [74]. Additionally, camelid VHH sequences share high homology with the human VH3 family, which further minimizes immunogenic potential and facilitates the design of humanized Nb derivatives [75], [76], [77]. It should be noted that the discussion about the low immunogenicity of Nbs usually stems from comparisons with traditional Abs. In fact, Nbs are not completely devoid of immunogenicity, and there may be significant differences in biochemical and immunological properties between naturally derived Nbs and their chemically synthetic counterparts (e.g., mirror-image proteins) [78], [79], [80]. Therefore, claims regarding this advantage need further evaluation in conjunction with specific experimental or clinical data related to the relevant Nbs.

#### 3.2.5 Structural compactness and engineering flexibility

The compact size of Nbs allows them to diffuse efficiently through tissue interstitium and traverse biological barriers, such as the blood-brain barrier (BBB), either naturally or through rational engineering [81], [82], [83]. The small molecular size also translates to rapid systemic clearance and a short plasma half-life, reducing non-specific background signals in therapeutic or imaging contexts [84]. Importantly, their simple and modular structure facilitates genetic fusion, multimerization, and fusion protein construction, while allowing high-level expression in both *Escherichia coli* (*E. coli*) and *Pichia pastoris* (*P. pastoris*) expression systems, thereby substantially lowering production costs [85].

## 4 Fabrication of nanobodies

Prior to experimental or clinical application, Nbs need to exhibit high binding specificity toward their target proteins; therefore, rigorous screening, optimization, and validation processes are essential to obtain Nbs that meet predetermined performance criteria [86].

### 4.1 Acquisition and screening of nanobodies

The generation of high-quality Nbs relies primarily on two key stages, the construction of Nb gene libraries and subsequent screening using in vitro surface display technologies.

#### 4.1.1 Construction of nanobody gene libraries

**Fig. 3:**
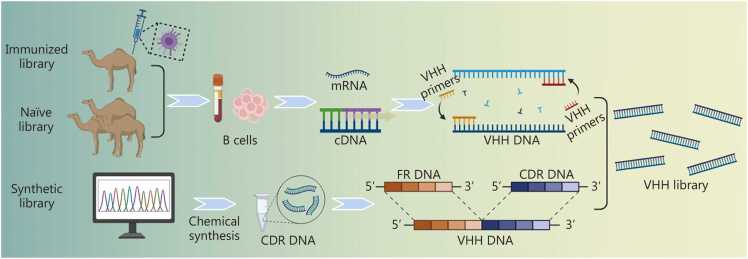
Construction of nanobody gene library. CDR. Complementarity-determining region; FR. Framework region; VHH. Variable domain of heavy chain of heavy chain antibody.

To obtain Nbs with high accuracy and practical applicability, it is essential to establish a reliable and high-quality Nb gene library. Currently, Nb libraries are mainly classified into two types, immune libraries derived from immunized animals and non-immune (naïve or synthetic) libraries constructed independently of antigen stimulation [87] **(**Fig. 3**)**.

##### 4.1.1.1 Immune library

Immune libraries are typically generated by immunizing camelids (e.g., camels or llamas) with one or more antigens. Healthy adult animals generally receive several injections of purified antigen over a period of approximately two months, depending on antigen characteristics and immune responsiveness. Following immunization, B lymphocytes are isolated from peripheral blood or lymphatic tissues, and total mRNA is extracted and reverse transcribed into cDNA. Using VHH-specific primers, the *VHH* gene segments are then amplified to construct the Nb gene library [88], [89], [90]. This strategy requires antigen-specific immune stimulation. Interestingly, heterologous immunization with multiple related antigens can generate an immune library that yields cross-reactive binding proteins capable of recognizing multiple variants [91]. Although the resulting libraries are relatively limited in size, the Nbs generated from them exhibit higher specificity and affinity, making them particularly suitable for the development of therapeutic Abs. Therefore, immune libraries remain the predominant method for Nb generation due to their reliability and effectiveness. The recently developed “LamaMouse” transgenic platform enables the generation of Nb immunization libraries in mice through the expression of camelid IgH heavy-chain genes. This strategy is expected to optimize and reduce the cost and complexity associated with Nb production [92].

##### 4.1.1.2 Non-immune (naïve and synthetic) library

Non-immune libraries are constructed through a similar molecular workflow but differ fundamentally in that they do not rely on antigen immunization. Instead, they are generated by directly isolating a diverse repertoire of *VHH* genes from the B cells of healthy animals or by rational synthetic design.

In naturally derived non-immune libraries, the diversity of the antibody library correlates with the number and variety of sampled B cell populations. Therefore, collecting large and heterogeneous blood samples from multiple animals is essential to ensure sufficient library complexity. This non-immunized approach avoids antigen-specific selection pressure during construction and enables the generation of libraries with exceptionally high capacity, often comprising up to 10⁹–10¹⁰ unique variants [93]. Such universal libraries can be reused for multiple targets and are particularly advantageous for screening Abs against poorly immunogenic or non-immunogenic antigens [94], [95]. However, in vitro affinity maturation is usually required to achieve Nbs with higher binding strength and improved functional properties [96].

Artificially designed synthetic libraries are based on known Nb structures and employ rational molecular engineering to expand diversity. In this approach, stable FRs are selected as the structural scaffold, while random mutations are introduced into the complementarity-determining regions to generate novel antigen-binding variants. The randomized CDR sequences are then assembled with the conserved FRs via polymerase chain reaction (PCR) or other molecular cloning techniques to construct full-length VHH sequences and form the final gene library [97]. One commonly used approach relies on oligonucleotides containing degenerate codons (e.g., NNK, where N = A/T/C/G and K = G/T) to synthesize combinatorial sequence variants using chemical methods, or introducing diversity by using predefined trinucleotides for synthesis, which allows for precise control of residue distribution and the elimination of termination codons [98], [99], [100]. In addition, synthetic libraries can also be built either by grafting natural Ab CDRs onto defined frameworks [101] or by using computer-designed CDRs that account for biophysical properties and contact‑surface amino acid interactions [102]. Synthetic library construction effectively overcomes natural immune diversification limits, allowing de novo design and targeted optimization of Nb properties. Thus, this approach is particularly well suited for the generation of engineered Abs tailored for specific biomedical or therapeutic applications [103], [104]. For instance, the recently developed synthetic Nb (sybody) technology, through the complete *in vitro* screening of synthetic libraries, can obtain 10–30 high-affinity binders with 500 pmol/L–500 nmol/L affinity for target proteins that are difficult to be used in immune libraries due to their high sequence conservation, toxicity or insufficient stability, within a short period of time. This fully demonstrates the efficiency and practicality of this strategy [105].

#### 4.1.2 *In vitro* surface display screening methods

Efficient screening of specific Nbs is a prerequisite for their research and application. In recent years, the efficiency of Nb screening has been greatly improved with the continuous development of *in vitro* display technologies **(**Table 1**)** [106], [107], [108], [109], [110], [111], [112], [113], [114], [115], [116], [117], [118], [119], [120], [121], [122], [123], [124], [125], [126], [127].

**Table 1:** Comparison of *in vitro* surface display screening methods for nanobodies (Nbs).

**Characteristic**	**Expression system**	**Screening method**	**Advantages**	**Disadvantages**	**Typical applications**	**References**
Phage display technology	Cell-free	Multiple rounds of panning	Large library capacity (10⁹–10¹¹); Short screening process; Low cost	High non-specific binding; Time-consuming; Suitable for low-molecular-weight proteins; Lack of post-translational modifications	Screening high-affinity antibodies (Abs)	[106], [107], [108], [109], [110], [111]
Bacterial surface display technology	Prokaryotic	Flow cytometric sorting	Moderate library capacity (10⁸–10¹⁰); Low cost and simple operation; Suitable for displaying small to medium-sized proteins	Lack of post-translational modifications	Rapid screening of Abs fit for expression in the prokaryotic system	[111], [112], [113], [114], [115], [116], [117]
Yeast surface display technology	Eukaryotic	Flow cytometric sorting	Correct molecular folding and glycosylation; Suitable for complex macromolecular proteins	Low library capacity (10⁷–10⁹); Time-consuming; Complex operation	Screening highly stable or Abs that require modification	[111], [118], [119], [120], [121]
Mammalian cell surface display technology	Eukaryotic	Flow cytometric sorting	Complete eukaryotic post-translational modification system; Protein expression with the native state	Low library capacity (10⁶–10⁸); High cost and time-consuming; Complex operation	Screening Abs requiring complex conformations or functions	[122], [123]
Ribosome/mRNA display technology	Cell-free	Multiple rounds of panning	Extremely large library capacity (10¹²–10¹⁴); Do not require hosts; Rapid operation; High screening efficiency	Lack of post-translational modifications; Complex operation; High cost	High-throughput screening and directed evolution	[110], [124], [125], [126], [127]

##### 4.1.2.1 Phage display technology

**Fig. 4:**
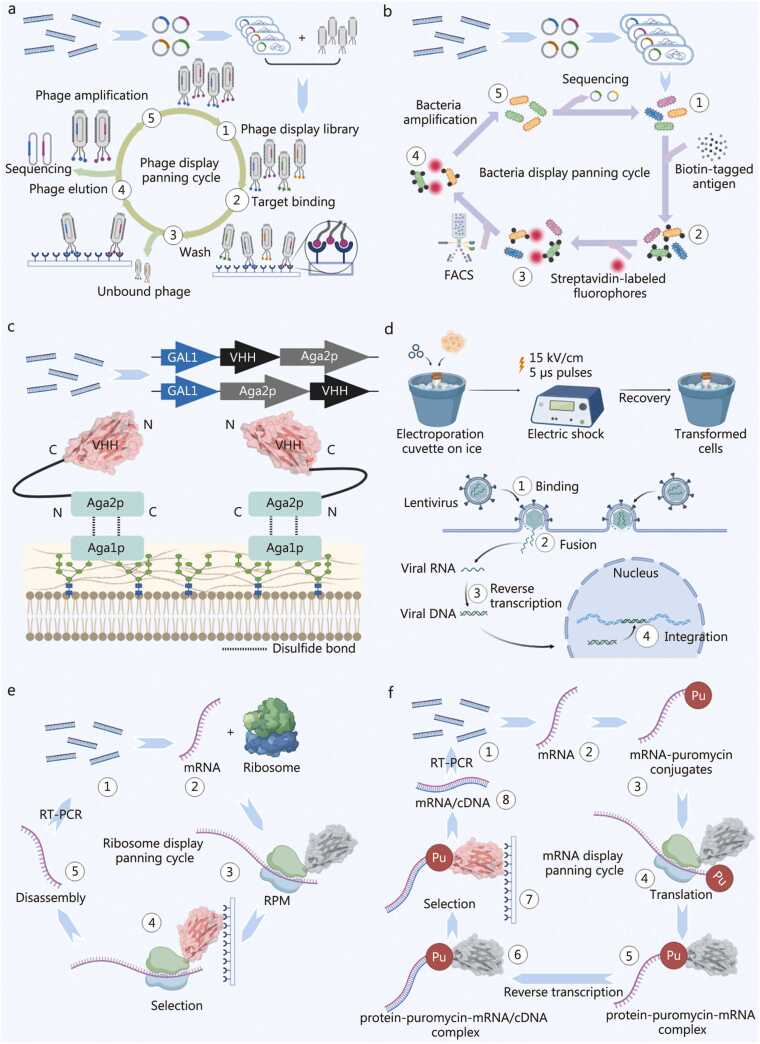
*In vitro* surface display technologies for nanobody (Nb) screening. **a** Schematic of phage display technology. **b** Schematic of bacterial surface display technology. **c** Schematic of yeast surface display of target proteins using the Aga1p-Aga2p system. **d** Schematic of electroporation for transient transfection and lentiviral transduction for stable transfection in mammalian cells. **e** Schematic of ribosome display technology. **f** Schematic of mRNA display technology. The protein structural data for VHH (PDB ID: 8FTG) used in panels c, e, and f of this figure are referenced from previously publication and visualized with PyMOL (v3.0.6) [22]. VHH. Variable domain of heavy chain of heavy chain antibody; PCR. Polymerase chain reaction; RT-PCR. Reverse transcription polymerase chain reaction; FACS. Fluorescence activated cell sorting; RPM. Protein-ribosome-mRNA.

Phage display is a classical and widely used strategy for screening antigen‑specific Nbs from immune libraries [106]. Through genetic recombination, the genes encoding Abs are fused to phage coat protein genes, enabling directional display of Ab fragments on the surface of progeny phages and thereby establishing highly diverse phage Ab libraries [107]. During the screening process, the phage library is incubated with immobilized antigens. Iterative panning steps are performed to progressively eliminate non‑specific binders while enriching phages that exhibit high affinity toward the target antigen. The retained phages are subsequently amplified by infecting *E. coli*. After 3–5 rounds of selection and enrichment, clones displaying strong and specific binding are significantly enriched. The corresponding high‑affinity Nb sequences are then identified through phage monoclonal isolation and DNA sequencing [108], [109] **(**Fig. 4**a)**. Phage display remains the most extensively applied technique for Nb screening. However, intrinsic limitations, such as reduced infectivity caused by the insertion of foreign genes, instability of fusion protein expression, and the difficulty of quantitative assessment of expression levels, may constrain its applicability in certain cases. Particularly, the screening process mainly enriches the clones with binding activity, but it may not effectively eliminate those candidates with high affinity but low expression levels or poor stability, leading to difficulties in the subsequent soluble expression and large-scale production of the screened Abs [110], [111].

##### 4.1.2.2 Bacterial surface display technology

Bacterial surface display is based on fusing the target protein gene with that of a carrier or anchor protein, followed by introduction of the recombinant construct into a bacterial host. Upon induction, the fusion protein is expressed and localized to the cell surface, achieving display of the target protein [112]. Various host bacteria can be employed in this technology, including *E. coli*, *Lactic acid bacteria*, *Pseudomonas putida*, and *Bacillus* species. Each host possesses distinct carrier proteins whose surface exposure and expression levels significantly influence display efficiency. Thus, careful selection of a compatible host-carrier combination is critical [113]. During screening, bacteria displaying the target protein on their surfaces are co-incubated with the antigen of interest. Fluorescent molecules are then used to label the antigen, and the positive clones are subsequently sorted using flow cytometry. Finally, plasmids are extracted from positively stained clones for sequence identification [114] **(**Fig. 4**b)**. Although bacterial systems are simple and cost-effective, they have several intrinsic limitations. (1) The constraints of bacterial transformation efficiency, along with the impact of the periplasmic/outer membrane environment on VHH folding and disulfide bond integrity, lead to a functional library size smaller than the theoretical diversity. (2) Display levels vary markedly between clones, leading to biased enrichment of highly expressed, stable binders rather than the highest affinity variants, and the small cell size limits the quantitative accuracy of flow cytometry-based affinity measurements. (3) The bacterial surface carries endotoxin and a variety of endogenous outer membrane components that can increase nonspecific binding and complicate work with complex or membrane-bound antigens. (4) The absence of eukaryotic post-translational machinery and the poor ability to mimic physiological selection pressures (e.g., in serum or at 37 ℃) reduce the predictive value of bacterial display for therapeutic developability, necessitating subsequent validation in yeast or mammalian systems [111], [115], [116], [117].

##### 4.1.2.3 Yeast surface display technology

Yeast surface display achieves expression of exogenous proteins on the yeast cell wall by fusing the target sequence to a native anchoring protein [118]. Common anchoring proteins include Aga1p, Aga2p, Cwp1p, Cwp2p, and Tip1p. Among these, the *Saccharomyces cerevisiae* Aga1p-Aga2p system is one of the most widely used. In this configuration, Aga2p serves as a soluble subunit covalently linked to the target protein, while Aga1p anchors to the cell wall and forms disulfide bonds with Aga2p, thereby immobilizing the fusion protein on the yeast surface [22], [119], [120] **(**Fig. 4**c)**. In practice, the target gene is inserted into a vector at a site distal to its functional domain to prevent interference with antigen binding. The constructions are then integrated into the yeast genome, ensuring stable expression. Signal peptides direct the secretion of the fusion protein to the cell surface, where the anchoring protein binds the cell wall matrix, presenting the Nb externally. The resulting yeast display library is incubated with fluorescently labeled antigens, and positive clones are isolated after several rounds of selection and flow cytometric sorting. Sequencing of enriched clones reveals the Nb sequences with desired binding properties [121]. Yeast display offers advantages such as high protein solubility, proper folding, and ease of screening. However, its library size is inherently limited by yeast transformation efficiency, which constitutes a notable drawback [111].

##### 4.1.2.4 Mammalian cell surface display technology

Mammalian surface display also involves both the construction of an Ab‑display library in mammalian cells and the subsequent screening of high‑affinity clones. In this system, exogenous Ab genes are introduced into mammalian cells (e.g., HEK293 or CHO) by fusion with a plasmid or viral vector encoding a transmembrane domain. Transient transfection allows short‑term display, whereas high‑titer lentiviral vectors enable stable and long‑term presentation on the cell membrane **(**Fig. 4**d)**. After establishing the display library, cells are incubated with fluorescently labeled antigens. Populations displaying strong binding signals are isolated via flow cytometric sorting, and the sequences of selected Nbs are determined by DNA analysis [122]. This platform offers substantial advantages, including proper protein folding, precise glycosylation, and expression of Ab molecules that closely mimic native structures found in higher eukaryotes. However, the technical complexity and high cost of mammalian display limit its use primarily to Nbs with intricate conformations or specialized functional requirements [123].

##### 4.1.2.5 Cell-free (ribosome and mRNA) display technologies

Recent advances in cell‑free protein synthesis systems have led to the development of ribosome and mRNA display, which provide powerful tools for in vitro selection from ultra‑large libraries [124], [125]. In ribosome display, termination codons are removed from Nb genes, allowing mRNA to remain complexed with the ribosome and nascent peptide during translation, thereby forming a stable protein-ribosome-mRNA (PRM) complex. This complex is then incubated with an immobilized antigen. After several selection rounds, bound complexes are isolated, and corresponding cDNA sequences are recovered via reverse transcription followed by PCR amplification to obtain high‑affinity Nb gene [22], [126] **(**Fig. 4**e)**. mRNA display operates on a similar principle but achieves genotype-phenotype linkage through the small molecule puromycin. Puromycin is chemically attached to the 3’‑end of the mRNA; during translation, it mimics an aminoacyl‑tRNA and forms a covalent bond with the nascent peptide’s C‑terminus, resulting in a stable mRNA-protein complex [22], [127] (Fig. 4**f**).

Both ribosome and mRNA display methods are independent of living host systems, thereby avoiding constraints such as host cell toxicity or proteolytic degradation of target proteins. They allow the generation of extremely large libraries (up to 10¹³ variants) and enable direct coupling between genotype and phenotype, resulting in high selection efficiency. Moreover, mutagenic PCR can be incorporated to facilitate in vitro directed evolution. Consequently, these technologies are expected to play an increasingly important role in the future development of high‑throughput display methodologies for Nb discovery [110].

### 4.2 Modification and optimization of nanobodies

**Fig. 5:**
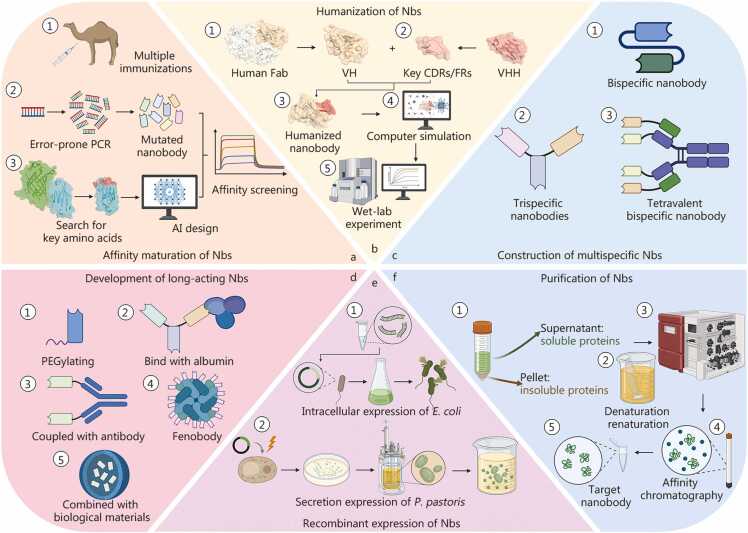
Strategies for nanobody (Nb) modification, optimization, and production. **a** Affinity maturation of Nbs. High-affinity Nbs can be obtained directly through ① multiple animal immunizations, or generated via ② error-prone PCR or ③ AI-assisted design to create variant libraries followed by screening. **b** Humanization of Nbs. Humanized Nbs are generated by grafting key CDRs/FRs onto a human Fab VH scaffold, followed by computer simulation and wet-lab validation. **c** Construction of multispecific Nbs. Engineering Nbs into bispecific or multispecific formats can significantly enhance their therapeutic potential. **d** Development of long-acting Nbs. Long-acting Nbs can be generated through ① PEGylation, ② albumin binding, or conjugation with ③ ④ long-lived proteins or ⑤ biomaterials. **e** Recombinant expression of Nbs. Nbs can be produced via ① intracellular expression in *E. coli* or ② secretory expression in *P. pastoris*. **f** Purification of Nbs. Soluble nanobodies ① are recovered directly from the supernatant, whereas insoluble nanobodies ② are refolded from the pellet via denaturation and renaturation; both recovery processes require purification by affinity chromatography. The protein structural data for the antigen-Nb complex (PDB ID: 8G0I), human Fab (PDB ID: 4PY7), and VHH (PDB ID: 8FTG) used in panels a and b of this figure are referenced from previous publications and visualized with PyMOL (v3.0.6) [9], [22], [128]. The structure of the humanized Nb was modeled using AlphaFold3 (https://alphafoldserver.com) and visualized with PyMOL (v3.0.6). CDR. Complementarity-determining region; FR. Framework region; VH. Variable region of heavy chain; VHH. Variable domain of heavy chain of heavy chain antibody; PCR. Polymerase chain reaction; AI. Artificial intelligence; *E. coli*. *Escherichia coli*; *P. pastoris*. *Pichia pastoris*; Fab. Fragment antigen-binding.

To enhance the affinity, stability, and functional performance of Nbs, targeted molecular modification and optimization are indispensable [9], [22], [128] **(**Fig. 5**)**.

#### 4.2.1 Affinity maturation of nanobodies

Affinity maturation refers to the natural process by which B cells, upon continual antigenic stimulation, undergo frequent mutations and selective expansion, thereby producing Abs with progressively increased binding affinity for their cognate antigens [129]. Because antigen-binding affinity is one of the most critical determinants of Nb efficacy, artificial affinity-maturation strategies are frequently employed when the binding performance of existing Abs is insufficient for downstream applications [130], [131].

Traditional in vitro random (blind) affinity maturation introduces random mutations into the CDRs, particularly CDR3, to generate diversified mutant libraries. The resulting variants are subjected to repeated rounds of antigen binding and elution. By progressively increasing selection pressure, such as lowering antigen concentration or enhancing washing stringency, variants with improved binding strength are preferentially enriched [132], [133]. In contrast, rational design-based affinity maturation relies on structural knowledge of the antigen-Ab interface. Key residues that modulate affinity or stability are identified using computational and AI-assisted tools, including homology modeling [134], molecular docking [135], molecular dynamics simulations [136], interface residue analyses [137], and mutational hotspot design [138]. Such in-silico approaches allow targeted optimization of biophysical parameters to improve Nb performance [139], [140]. Recently, the AI-based IsAb2.0 Ab design model has successfully provided five mutation schemes to enhance the binding affinity of the humanized Nb J3 (HuJ3) targeting human immunodeficiency virus type 1 (HIV-1) envelope glycoprotein gp120 [141]. Notably, higher affinity does not always translate into better functional outcomes; excessively strong binding may hinder antigen dissociation and reduce biological efficacy. Therefore, affinity maturation must balance affinity, specificity, and stability to yield Nbs optimally suited for specific applications [142].

#### 4.2.2 Humanization of nanobodies

When exogenous Abs are administered to humans, severe immune rejection or hypersensitivity may occur. Although Nbs exhibit markedly lower immunogenicity than traditional monoclonal Abs, several studies have detected anti-drug Abs (ADAs) against Nbs in patients treated with Nb-based therapies [143], [144], [145]. Therefore, humanization remains essential to further improve their safety, efficacy, and clinical viability [6], [146].

Ab humanization involves genetically engineering animal-derived Abs so that most or all non-human residues are replaced with human equivalents, thereby minimizing immunogenicity while maintaining binding specificity and affinity [147]. For VHHs, this process typically begins by determining the three-dimensional structure via X-ray crystallography, nuclear magnetic resonance (NMR) spectroscopy, or computational modeling (e.g., AlphaFold, Rosetta). Subsequent functional mapping identifies the antigen-contacting residues within the CDRs and the conserved residues in FRs responsible for structural integrity. A human VH framework with the highest sequence similarity to the camelid VHH is then selected, and non-human residues are substituted accordingly while preserving the CDR structures. Computational modeling, molecular docking, and molecular dynamics simulations are used to predict antigen-binding modes of the humanized variants. Based on these models, further sequence optimization can be performed to retain or enhance binding affinity. The final constructs are experimentally validated for antigen recognition, structural stability, and immunogenicity *in vitro* and *in vivo* [148], [149], [150], [151].

A recent study indicated that the thermally stable FR variants obtained by screening the human VH library can be fused with the diverse CDRs of VHH to construct a human VH sdAb library. This hybrid approach facilitates humanization while preserving native binding capabilities, offering a versatile platform for developing various applicable Nbs [76].

#### 4.2.3 Construction of multispecific nanobodies

The construction of multispecific Nbs has emerged as a promising direction for maximizing Nb therapeutic potential. Owing to their compact structure, high solubility, and ease of genetic engineering, multiple Nb units can be fused into a single multispecific molecule without mutual interference [152]. One key functional advantage of such constructs is avidity, the cumulative binding strength resulting from the simultaneous engagement of multiple binding domains with their respective epitopes. Unlike affinity, which measures the strength of a single binding site, avidity incorporates the effects of multivalency and spatial arrangement, leading to greater overall binding stability and reduced dissociation rates [153], [154]. In multispecific Nbs, enhanced avidity can translate into improved target retention, stronger neutralizing potency, and prolonged *in vivo* half-life [155], [156]. Moreover, multispecific Nbs can simultaneously target multiple signaling pathways or distinct epitopes on the same antigen, thereby minimizing the risk of drug resistance and enhancing therapeutic efficacy [157], [158]. Both in vitro and *in vivo* assembly strategies have been employed, such as flexible peptide linkers, Ab-domain fusion, coiled-coil-mediated self-assembly, chemical conjugation, and hydrophobic self-association, to generate multivalent or multispecific constructs [159]. For example, replacing two Nbs targeting the same antigen with domains corresponding to the VH and VL regions of the human V_H_3-23 variant can produce a bispecific tetra-Nb capable of potent neutralization. This modular workflow can be extended to a wide range of protein targets, providing a generalizable framework for designing multispecific molecules [160].

#### 4.2.4 Development of long-acting nanobodies

Several strategies have been developed to extend the systemic persistence of Nbs. Chemical modification through PEGylation can increase hydrodynamic size and reduce renal clearance [161]. Alternatively, Nbs can be fused or complexed with long-lived endogenous serum proteins (e.g., albumin) or cells to exploit physiological recycling mechanisms [162], [163]. In particular, myelin oligodendrocyte glycoprotein (MOG)‑binding Nbs have been used to extend the effective duration of protein therapeutics in the central nervous system, offering potential advantages for treating neurodegenerative disorders [164]. Furthermore, conjugation to Ab Fc domains can reconstitute Fc-mediated effector functions and prolong systemic circulation [165], [166]. Another representative example is the “fenobody”, a ferritin-Nb fusion that not only increases apparent binding affinity but also markedly improves pharmacokinetic stability [167]. Integration of Nbs with biocompatible materials also offers half-life benefits. For instance, encapsulating an anti-vascular endothelial growth factor (VEGF)  Nb within niosome nanoparticles (NNPs) substantially prolongs its circulation time to a certain extent [168]. Together, these strategies enhance pharmacokinetic performance, reduce dosing frequency, and improve patient compliance, providing a crucial guarantee for the clinical translation of Nb.

### 4.3 Large-scale production of nanobodies

Efficient large-scale production of Nbs involves two key stages, recombinant expression and purification **(**Fig. 5**)**.

#### 4.3.1 Recombinant expression of nanobodies

Recombinant Nb production commonly utilizes both prokaryotic and eukaryotic expression systems [169]. *E. coli*-based systems are favored for their well-established genetics, rapid growth, short production cycles, low cost, and suitability for large-scale fermentation [170]. Recombinant expression can occur either in the cytoplasm or the periplasmic space [171]. Because Nbs contain at least one canonical disulfide bond, the reducing cytoplasmic environment can disrupt proper disulfide formation, leading to misfolded proteins or insoluble inclusion bodies [172], [173]. In such cases, refolding procedures involving denaturation and renaturation or fusion with solubility-enhancing tags, such as small ubiquitin-like modifier (SUMO), are necessary to obtain bioactive products [174]. Interestingly, a study has reported that certain Nbs themselves can act as solubilization chaperones, improving the soluble expression of aggregation-prone target proteins [175]. In contrast, the periplasmic compartment provides an oxidative environment conducive to correct disulfide bond formation and is rich in molecular chaperones that promote accurate folding. For periplasmic secretion, a signal peptide is typically fused to the Nb’s N-terminus to mediate translocation via the general secretory (Sec), signal recognition particle (SRP), or twin-arginine translocation (Tat) pathways [176]. Several studies have optimized this process by supplementing cultures with vitamins to enhance recombinant yield [177] or by adding agents such as Triton X-100 and glycine to promote secretion of soluble Nbs into the extracellular milieu [145]. Additionally, fusion of Nbs with superfolder GFP (sfGFP) tags or alternative signal peptides has also been shown to improve secretion efficiency without cell lysis, simplifying downstream processing [178], [179], [180]. Notably, direct *in situ* delivery approaches have been described in which engineered probiotic *E. coli* secrete therapeutic Nbs in the intestinal tract, providing a potential strategy for oral administration [181]. Despite these advantages, the *E. coli* expression system presents several inevitable drawbacks, such as endotoxin contamination and the absence of post-translational modifications, both of which may affect the structural integrity or biological activity of Nb products [182].

Yeast expression systems, particularly *P. pastoris*, enable correct folding, disulfide formation, and limited glycosylation while remaining free of lipopolysaccharides (LPS) contamination [183]. The system supports high-density fermentation and employs the strong methanol-inducible AOX1 promoter, which facilitates tight transcriptional regulation and high-level expression of heterologous proteins [184]. Typically, exogenous genes are linearized and integrated into the yeast genome, ensuring stable inheritance and preventing plasmid loss, thereby improving consistency and yield [185], [186]. Furthermore, *P. pastoris* supports secretory expression, allowing direct recovery of Nbs from the culture supernatant and greatly simplifying purification steps. However, the presence of non-human high-mannose glycosylation patterns in yeast products can induce immunogenicity and thus remains a challenge for therapeutic development [187].

#### 4.3.2 Purification of nanobodies

For purification, high-affinity and specific affinity chromatography systems have long been employed to isolate Abs from complex mixtures [188]. Genetic engineering approaches enable the fusion of affinity tags, most frequently a 6× His tag, to the N- or C-terminus of Nbs, facilitating purification via nickel-based matrices such as HisTrap®, Ni-NTA, or Ni-Sepharose resins [189], [190]. However, in some cases, enzymatic tag removal may be required, potentially introducing impurities or increasing production costs. To overcome these limitations, alternative strategies employing mixed-mode cation exchange chromatography resins, such as Capto multimodal cation exchanger (MMC) and Eshmuno hydrophobic cation exchanger (HCX), have been explored, achieving yields of up to 84.5% and purities of up to 99.2% [191], [192]. In addition, size-exclusion chromatography is often employed for further purification to improve protein purity and homogeneity [193].

## 5 Applications of nanobodies

**Fig. 6:**
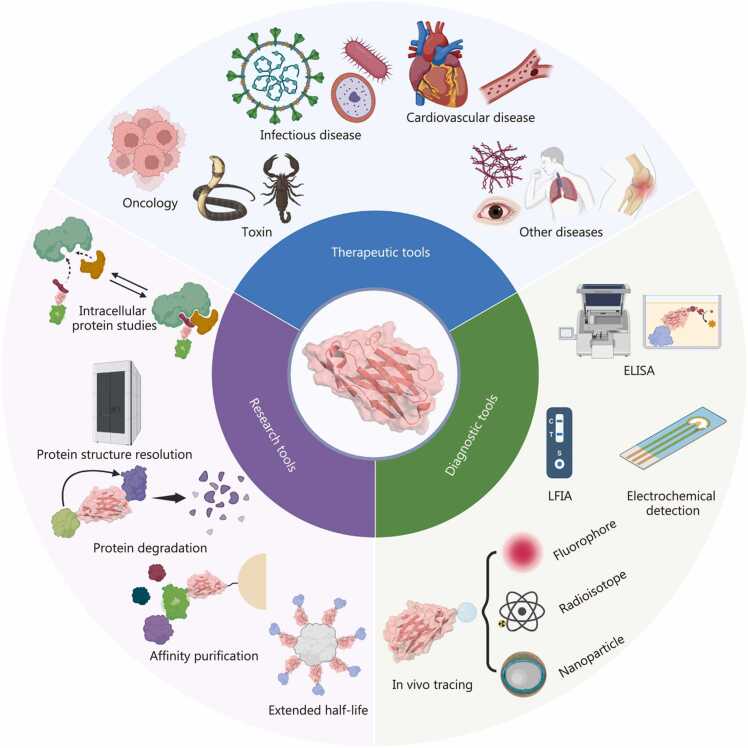
Application of nanobodies (Nbs) in therapeutics, diagnostics, and research. The protein structural data for VHH (PDB ID: 8FTG) is referenced from previously publication and visualized with PyMOL (v3.0.6) [22]. VHH. Variable domain of heavy chain of heavy chain antibody; ELISA. Enzyme-linked immunosorbent assay; LFIA. Lateral flow immunoassay.

Nbs, owing to their unique physicochemical and biological properties, have garnered significant attention and have been widely applied in therapeutics, diagnostics, and basic research, with promising prospects for future development [22] **(**Fig. 6**)**.

### 5.1 Nanobodies as therapeutic tools

#### 5.1.1 Approved nanobody therapeutics

In recent years, a variety of drugs based on Nbs have successfully been approved for clinical use **(**Table 2**)** [194], [195], [196], [197], [198], [199], [200], [201], [202], [203], [204], [205], [206], [207], [208], [209], [210], [211], [212], [213], [214], [215], [216], [217], [218], [219], [220].

**Table 2:** Clinical research progress of Nb-based drugs.

**Drug name**	**Targets**	**Disease**	**Status/Phase**	**Clinical trial registration number**	**Approval date/Study start date**	**References**
Caplacizumab (Cablivi)	Vascular von Willebrand factor ligand	Acquired thrombotic thrombocytopenic purpura	Approved	NA	August 30, 2018	[194]
Envafolimab (KN035)	Programmed death-ligand 1	Advanced solid tumors	Approved	NA	November 24, 2021	[195]
Ciltacabtagene autoleucel (cilta-cel)	B-cell maturation antigen	Relapsed/refractory multiple myeloma	Approved	NA	February 28, 2022	[196]
Ozoralizumab (Nanozora)	TNF-α; Albumin	Rheumatoid arthritis	Approved	NA	September 26, 2022	[197]
Sonelokimab (M1095)	IL-17A; IL-17F	Hidradenitis suppurativa	Phase III	NCT07007637	June 27, 2025	[198]
Gefurulimab (ALXN1720)	Complement component 5	Myasthenia gravis	Phase III	NCT06607627	November 13, 2024	[199]
LMN-201	Clostridioides difficile toxin B	Clostridioides difficile infection	Phase III	NCT05330182	August 29, 2024	[200]
Erfonrilimab (KN046)	Cytotoxic T-lymphocyte-associated protein 4; Programmed cell death ligand 1	Advanced solid tumors	Phase III	CTR20212934	December 3, 2021	[201]
Brivekimig (SAR-442970)	Tumor necrosis factor ligand superfamily member 4; TNF-α ligand	Type 1 diabetes; Hidradenitis suppurativa; Immunodysregulation disorders; Ulcerative colitis	Phase II	NCT06812988	February 28, 2025	[202]
ZL-1102	IL-17A	Plaque psoriasis	Phase II	NCT06380907	May 22, 2024	[203]
HLX-53	Immunoglobulin and ITIM domain protein T-cell immunoreceptor	Advanced solid tumors; Malignant lymphoma	Phase II	NCT06349980	August 5, 2024	[204]
Lunsekimig (SAR-443765)	IL-13 receptor; Thymic stromal lymphopoietin	Asthma	Phase II	NCT06102005	October 16, 2023	[205]
trovocabtagene autoleucel (C-CAR088)	B-cell maturation antigen	Multiple myeloma	Phase II	NCT05521802	November 11, 2022	[206]
LAVA-1207	Prostate-specific membrane antigen; T-cell receptor	Hormone-resistant prostate cancer	Phase II	NCT05369000	January 17, 2022	[207]
LMN-101	*Campylobacter jejuni* flagellin	*Campylobacter jejuni* infection	Phase II	NCT04182490	February 21, 2022	[208]
[^131^I]-SGMIB Anti-HER2 VHH1	Human epidermal growth factor receptor 2	Breast cancer	Phase II	NCT04467515	September 14, 2021	[209]
BI-836880	Vascular endothelial growth factor A ligand; Angiopoietin-2	Wet age-related macular degeneration	Phase II	NCT03861234	June 27, 2019	[210]
Gontivimab (ALX-0171)	Respiratory syncytial virus F protein	Respiratory syncytial virus infection	Phase II	NCT02979431	January 11, 2017	[211]
V565	TNF-α ligand	Crohn’s disease; Ulcerative colitis	Phase II	NCT02976129	December, 2016	[212]
Vobarilizumab (ALX-0061)	IL-6 receptor; Albumin	Rheumatoid arthritis	Phase II	NCT02309359	January, 2015	[213]
RC1416	IL-4 receptor; IL-5 receptor subunit α	Moderate to severe asthma	Phase I	NCT06911866	October 25, 2025	NA
LCAR-AIO	B-lymphocyte antigen CD19; B-lymphocyte antigen CD20; B-lymphocyte adhesion molecule	Systemic lupus erythematosus; B-cell lymphoma	Phase I	NCT06869278	June 17, 2025	[214]
ATTO-1310	IL-31	Atopic dermatitis	Phase I	NCT06787586	January 14, 2025	NA
PF-08046052 (SGN-EGFRd2)	Epidermal growth factor receptor; T-cell receptor gene	Solid tumors	Phase I	NCT05983133	November 14, 2023	[215]
LQ-043H	Thymic stromal lymphopoietin	Respiratory system diseases	Phase I	CTR20230092	January 13, 2023	[216]
NM-01	Programmed cell death ligand 1	Metastatic non-small cell lung cancer	Phase I	NCT04992715	May 3, 2022	[217]
LQ-036	IL-13 receptor; IL-4 receptor	Asthma; Chronic obstructive pulmonary disease	Phase I	NCT04993443	September 6, 2021	[218]
KN044	Cytotoxic T-lymphocyte-associated protein 4	Malignant neoplasms	Phase I	NCT04126590	January 9, 2019	NA
M6495	ADAMTS5; Albumin	Osteoarthritis	Phase I	NCT03224702	September 4, 2017	[219]
BI-655088	CX3C chemokine receptor 1	Chronic kidney disease	Phase I	NCT02696616	March 16, 2016	[220]

Information was obtained from ClinicalTrials.gov (https://clinicaltrials.gov/) and the Chinese Clinical Trial Registry (https://www.chictr.org.cn/), with data current as of October 31, 2025. ITIM. Immunoreceptor tyrosine-based inhibitory motif; NA. Not available; TNF-α. Tumor necrosis factor-α; IL. Interleukin

Caplacizumab (trade name: Cablivi) is the world’s first Nb drug to be approved for market. It consists of two identical humanized building blocks that are genetically linked by a three-alanine linker. Caplacizumab can bind to the A1 domain of von Willebrand factor (vWF) with high specificity and high affinity, thereby inhibiting the interaction between vWF and the platelet membrane glycoprotein GP1b-IX-V receptor, effectively preventing abnormal platelet aggregation and microthrombosis. It shows significant efficacy in the treatment of acquired thrombotic thrombocytopenic purpura (aTTP), and is also friendly to children. Since its approval in 2018, the clinical application of caplacizumab has fully verified the effectiveness and safety of Nbs as therapeutic protein drugs [221], [222], [223].

Envafolimab is the world’s first approved subcutaneously (SC) administered programmed death ligand 1 (PD-L1) inhibitor. This drug is constructed by fusing a humanized anti-PD-L1 Nb with the Fc fragment of human IgG1. It can bind to PD-L1 on the surface of tumor cells or immune cells with high affinity and specificity, blocking the interaction between PD-L1 and its receptor PD-1, thereby relieving its inhibition on the immune activity of T cells and restoring the body’s own anti-tumor immune response. Clinically, envafolimab is used to treat adult patients with previously treated microsatellite instability-high (MSI-H) or deficient mismatch repair (dMMR) advanced solid tumors [195], [224].

Ciltacabtagene autoleucel (cilta-cel) was approved in 2022 for patients with relapsed/refractory multiple myeloma (RRMM). It is a chimeric antigen receptor T-cell (CAR-T) therapy targeting B-cell maturation antigen (BCMA). By genetically engineering the patient’s own T cells, two Nbs specific to BCMA are introduced onto their surface to enable them to recognize and kill malignant cells, thereby achieving the therapeutic effect. Its approval indicates that the application of Nb technology has successfully expanded beyond traditional antibody-based therapeutics into the cutting-edge field of cell therapy, underscoring its substantial potential as a modular targeting component [225], [226], [227].

Ozoralizumab is the world’s first approved trivalent bispecific Nb drug, used for the treatment of rheumatoid arthritis (RA). Its structure comprises two tumor necrosis factor-α (TNF-α)-targeting Nbs and one human serum albumin (HSA)-targeting Nb. The HSA-binding domain can reversibly associate with circulating albumin, significantly decreasing renal clearance and thereby extending the dosing interval to once a month, which substantially enhances patient convenience [197], [228], [229].

#### 5.1.2 Preclinical research

##### 5.1.2.1 Tumor therapy

Compared with conventional Abs, Nbs possess a smaller molecular size, enhanced tissue penetration, and high specificity for target molecules, enabling them to access tumor microenvironments that are often inaccessible to larger Ab therapeutics [230]. Construction of bivalent or trivalent multimeric Nbs further enhances their affinity for the target [231]. Importantly, their lack of Fc domains also results in substantially reduced immunogenicity [25], positioning Nbs as attractive agents for tumor-targeted therapy [232]. One notable target is the epidermal growth factor receptor (EGFR), which is pivotal in epithelial tissue development and cancer progression [233]. Bispecific Nbs targeting the dimerization interface of EGFR have demonstrated potent inhibition of tumor growth *in vitro* and *in vivo*, effectively overcoming resistance commonly encountered in EGFR-targeted therapies [234]. Likewise, bispecific Nbs targeting both EGFR and insulin-like growth factor-1 receptor (IGF-1R) have shown remarkable efficacy in pancreatic cancer models [235].

Nb-mediated targeted drug delivery can further enhance treatment safety and efficacy [236]. For example, fusing interleukin-2 (IL-2) with Nbs specific for the extra domain B (EIIIB) of fibronectin on tumor cells and administering this fusion protein intravenously in murine models substantially improved therapeutic outcomes [237]. Researchers have also developed a one-step method to prepare immunoliposomes for targeted therapy by self-assembling chimeric Nbs (cNbs), comprising anti-human epidermal growth factor receptor 2 (HER2) Nbs, flexible peptide linkers, and hydrophobic single-pass transmembrane domains, onto liposomes. Results indicated that up to 2500 cNbs could be anchored on the liposomal membrane without causing steric hindrance, and drug-loaded immunoliposomes exhibited a 10- to 20-fold increase in cytotoxicity against HER2-overexpressing cancer cells [238]. Recently, cell-free protein synthesis systems have emerged as a powerful platform for the rapid production of immunoliposomes. Using these approaches, multiple types of functional binding proteins, such as Nbs, computationally designed proteins, and therapeutic peptides, have been successfully expressed and conjugated to liposomes. These cell-mimetic nanoparticles are expected to be widely applied in cancer therapy and drug development [239], [240].

The Nb-based CAR-T therapy is also gaining prominence [241]. A Phase I clinical study utilizing NS7CAR T-cells employing Nb-based targeting domains demonstrated promising efficacy and safety in patients with CD7-positive acute myeloid leukemia (AML) [242]. Given the heterogeneity of antigen expression (e.g., CD123 and CD33) among AML patients, bispecific CD33/CD123 Nb T-cell engagers (CD33/CD123-TCE) have been devised to overcome the limitations of single-targeted therapeutics, resulting in improved efficacy and broader patient applicability [243].

##### 5.1.2.2 Toxin therapy

Nbs are particularly well-suited for use as antidotes in toxin neutralization therapies [244], [245]. In contrast to traditional antitoxin sera, which are associated with high immunogenicity and production cost [246], Nbs offer a more efficient and scalable platform for toxin neutralization [247].

Venomous snakebite, chiefly from North American coral snakes and Bothrops species, remains a critical clinical challenge. Mixtures of Nbs screened via phage display have provided cross-neutralization against coral snake toxins in murine models, mitigating lethality and demonstrating synergy when combined to counteract distinct venom components [248], [249], [250], [251]. Similarly, bispecific and combinatorial Nb therapeutics have proven effective in neutralizing potentially fatal scorpion venoms in animal studies, often exhibiting synergistic protection [252], [253].

##### 5.1.2.3 Treatment of infectious diseases

Infectious diseases continue to pose a major global health burden. As next-generation Ab tools, Nbs can be engineered for specificity and multivalency to directly target the epitopes of pathogen including viruses, bacteria, fungi, and parasites [254], [255].

In antiviral applications, Nbs have shown efficacy by binding viral surface proteins and blocking cellular entry [256]. For example, as a highly infectious virus, severe acute respiratory syndrome coronavirus 2 (SARS-CoV-2) has been the target of several Nbs that bind to its surface receptor-binding domain (RBD) and inhibit its interaction with angiotensin-converting enzyme 2 (ACE2), thereby effectively mitigating disease progression [257], [258]. The adaptive multi-epitope targeting with enhanced avidity (AMETA) platform, which conjugates bispecific Nbs to an IgM scaffold, has achieved more than a million-fold increase in neutralization potency compared to monomeric Nbs [259]. Nbs targeting the CD4 binding site of HIV have also shown significant promise as broad-spectrum neutralizers [260], [261], [262]. For norovirus, dimeric fusion proteins (e.g., Fc-Nb26) have enabled broad-spectrum cross-reactivity and effective infection blockade [263].

Nbs can also reduce bacterial adhesion and mask bacterial toxins, offering potential alternatives to combat antimicrobial resistance [264], [265]. For example, anti-F17 Nbs hinder *E. coli* attachment and prevent severe diarrheal disease in livestock [266], [267]. Invasive fungal diseases pose a significant threat to global health security. Nbs targeting β-1,3-glucanosyltransferase (Gel) demonstrate antifungal properties through disruption of vital cell wall biosynthetic processes [268]. In parasitology, targeting of *Plasmodium falciparum* surface protein mosquito midgut screen 43 (PIMMS43) by specific Nbs has exhibited striking efficacy in blocking malarial transmission [269], [270]. The innovative approach of deploying transgenic mosquitoes expressing these Nbs, potentially via gene-drive technology, holds promise for sustained vector-control strategies.

##### 5.1.2.4 Treatment of circulatory system diseases

Nbs have demonstrated broad therapeutic utility in circulatory diseases, functioning as specific inhibitors of enzymes and receptors implicated in pathogenesis [271]. In light chain amyloidosis (AL), complexation of Nbs with amyloidogenic light chains inhibits cardiac deposition and toxicity [272]. Nbs targeting the angiotensin II type 1 receptor (AT1R) act as effective antagonists comparable to angiotensin receptor blockers (ARBs), with particular safety advantages in special populations such as pregnant women [273]. In preclinical thrombosis models, an Nb-Fc fusion capable of recognizing FXII and inhibiting its activation has shown efficacy in blocking arterial thrombosis without impacting normal coagulation, and may further benefit management of inflammatory vasculopathies and extracorporeal membrane oxygenation (ECMO)-associated thrombosis [274].

##### 5.1.2.5 Treatment of other diseases

Beyond oncology, toxins, infectious and circulatory diseases, Nbs have found application in the treatment of central nervous system disorders, ocular pathologies, musculoskeletal conditions, and respiratory diseases. For Alzheimer’s disease, Nbs directed against key sites on Tau and Aβ proteins, such as Z70, targeting PHF6 of Tau, can inhibit pathogenic aggregation and mitigate neurodegeneration [275], [276]. Fusion of Tau-specific Nbs to E3 ligase domains (e.g., from TRIM21) has enabled targeted degradation of pathological Tau protein aggregates [277]. Moreover, multivalent Nb constructs incorporating reactive oxygen species (ROS)-scavenging scaffolds have further broadened the disease-treating potential by simultaneously limiting Aβ pathology and oxidative stress [278]. In ophthalmology, natamycin-conjugated Nbs against fungal β-glucans have demonstrated efficacy in treating fungal keratitis by disrupting biofilm formation and fungal proliferation [279], [280]. For osteoarthritis, the anti-ADAMTS-5 Nb M6495 has shown promising results in Phase I clinical trials, inhibiting glycan degradation and protecting cartilage, with good safety and tolerability [219], [281]. Finally, Nbs’ stability and inhalability enable novel strategies for respiratory disease therapies, exemplified by the bivalent LQ036 Nb targeting IL-4Rα, which exhibits favorable pharmacokinetics and tissue distribution profiles in humanized mouse models, highlighting its clinical promise as an inhaled biologic therapeutic for asthma [282].

### 5.2 Nanobodies as diagnostic tools

Owing to their high specificity, stability, ease of genetic and chemical modification, and robust performance in diverse environments, Nbs represent a new generation of diagnostic reagents with transformative potential. Their applications span in vitro diagnostic (IVD) systems and *in vivo* molecular imaging, providing both precision and versatility in disease detection.

#### 5.2.1 *In vitro* diagnostics

Nbs can be integrated into a wide range of IVD assays. Among them, the enzyme-linked immunosorbent assay (ELISA) remains one of the most widely adopted techniques due to its simplicity, sensitivity, and reproducibility [283]. Incorporation of Nb technology has further enhanced these attributes. For instance, fusion of horseradish peroxidase (HRP) with Nbs has been successfully employed in competitive ELISAs for the sensitive, specific, and stable detection of Abs against African swine fever virus (ASFV) [284]. In another innovation, Nbs fused with ferritin form multivalent self-assembling nanostructures, which organize into 24-mer cage-like assemblies. This multivalent platform exhibits up to a 35-fold increase in binding capacity to *Salmonella* antigens compared with monomeric counterparts, markedly improving the sensitivity of ELISA detection systems [285].

Nbs have also advanced lateral flow immunoassays (LFIAs) [286]. For example, an LFIA employing Nbs specific for trypanosome pyruvate kinase (TcoPYK) significantly improved diagnostic specificity for *Trypanosoma congolense* infection in livestock [287]. Similarly, through the integration of two orthogonal protein-docking systems, SpyTag/SpyCatcher and Im7/CL7, a “double-Y” Nb assembly was constructed with notably enhanced antigen affinity. When applied to LFIA for SARS-CoV-2 nucleocapsid protein (N-protein) detection, this system achieved sensitivity as low as 500 pg/ml, underscoring its outstanding diagnostic performance [288].

In the field of biosensing, Nbs function effectively as recognition elements on sensor surfaces. Upon binding to target analytes, Nb-coated sensors exhibit changes in surface potential or charge density measurable via field-effect transistors (FETs), enabling real-time label-free detection [289]. A representative example is an electrochemical competitive immunosensor incorporating Nbs for detecting 3-phenoxybenzoic acid (3-PBA), a biomarker of pyrethroid exposure, demonstrating potential for human toxicological screening [290]. Additional examples include a ratio-based bioluminescence resonance energy transfer (BRET) nano Q-body, which allows rapid quantification of the anticancer drug methotrexate (MTX). This device can be lyophilized onto paper for portable, low-cost testing, highlighting the compatibility of Nb assays with point-of-care diagnosis [291]. Human toxocariasis (HT) is caused by the larvae of *Toxocara canis*. Due to its low concentration in the blood, traditional detection methods are difficult to achieve high sensitivity detection [292]. Nb-based electrochemical sensors have also achieved remarkable sensitivity in parasite detection. For instance, one-step detection of human *Toxocara canis* antigens utilizing Nb electrodes provided ultrasensitive and highly specific measurement, eliminating cross-reactivity with other nematode antigens and representing one of the most efficient diagnostic strategies reported to date [293]. Furthermore, a sandwich-type immunosensor integrating Nbs and LiSmZrO₃ perovskite nanoparticles has been developed for the detection of the tumor biomarker macrophage-capping protein (CapG). This sensor displayed a broad dynamic range (0–1200 pg/ml) and a remarkably low detection limit (326.4 pg/ml in phosphate-buffered saline), confirming its promise for clinical onco-biomarker monitoring [294].

#### 5.2.2 *In vivo* tracing

In vivo imaging applications capitalize on Nbs’ unique advantages, robust thermal and chemical stability, facile conjugation with fluorescent dyes or radionuclides, high target specificity, and small molecular size that allows rapid tissue penetration and clearance through the liver [295], [296], [297]. These features render Nbs ideal molecular tracers for both fluorescence and nuclear imaging.

Fluorescence molecular imaging provides real-time intraoperative visualization of tumors, aiding complete resection and improving surgical outcomes. However, conventional fluorophores lack specificity for malignant tissue [298]. Nb-based probes overcome this limitation. For instance, near-infrared (NIR) fluorophore s775z-labeled Nbs targeting urokinase-type plasminogen activator receptor (uPAR), a molecule overexpressed in numerous tumors, exhibited rapid tumor accumulation and high contrast within 1 h post-injection, greatly outperforming non-targeted controls. Success in the *in situ* human glioma models highlights its promise for fluorescence-guided surgery [299].

Radiolabeled Nbs also serve as precise tools for non-invasive nuclear imaging of tumor biomarkers. For example, a trophoblast cell surface antigen 2 (Trop2)-specific Nb (MY6349) labeled with technetium-99m (^99m^Tc) yielded the molecular probe [^99m^Tc] Tc-MY6349, which enabled real-time visualization of Trop2-positive breast cancer lesions in patients through single photon emission computed tomography/computed tomography (SPECT/CT) imaging. Within 15 min of tracer administration, heterogeneous Trop2 expression was clearly detectable in both primary and metastatic foci, while rapid clearance from non-tumor tissues resulted in excellent tumor-to-background contrast [300].

Nbs can further be engineered into nanoparticle- or microsphere-based contrast agents for ultrasound and multimodal imaging applications [301]. For example, vascular cell adhesion molecule 1 (VCAM-1), a key mediator in atherosclerosis via monocyte recruitment to arterial walls, has been targeted using VCAM-1-specific Nbs linked to microsphere contrast agents through maleimide-thiol chemistry. This strategy minimizes Ab immunogenicity and biotin-related artifacts. In atherosclerotic mouse models, contrast-enhanced ultrasound molecular imaging (CEUMI) demonstrated significantly higher signal intensities in diseased regions, corroborated by validation in human carotid endarterectomy samples, thus establishing a foundation for clinical translation [302], [303].

### 5.3 Nanobodies as research tools

Nbs have become indispensable reagents in modern biomedical research owing to their unique biochemical characteristics, allowing them to function not only as structural and analytical probes, but also as molecular regulators for investigating and modulating protein function in living systems.

#### 5.3.1 Prolonging drug serum half-life

In drug development, serum half-life is a pivotal pharmacokinetic parameter influencing therapeutic efficacy and dosage regimen [304]. The above has already introduced the strategies for optimizing the pharmacokinetic properties of Nbs themselves. Furthermore, Nbs can also serve as molecular tools to help other drugs prolong their half-life in the body.

Albumin, a major plasma protein, binds a wide array of endogenous and exogenous molecules and thus modulates their circulation and metabolism [305]. Therefore, Nbs, particularly those engineered to bind serum albumin, can be conjugated to biologics or small molecules to prolong their systemic exposure, thereby enhancing efficacy and reducing dosing frequency. A clinically validated example is ozoralizumab, which supports once-monthly administration [306]. Similarly, after fusion with an anti-HSA Nb, the sdAb-drug conjugate (sdADC) against the oncofetal antigen 5T4 increased its half-life by 10-fold in wild-type mice and 5-fold in xenograft models, accompanied by stronger tumor accumulation and lower hepatotoxicity [307]. These data demonstrate that HSA-specific Nbs provide a robust and versatile platform for pharmacokinetic optimization of therapeutic agents.

#### 5.3.2 Nanobodies as affinity capture reagents

Nbs are widely utilized as affinity ligands for purification and detection. Anti-adenovirus Nbs have proven effective for one-step immuno-affinity purification of viral vectors, maintaining performance over >2000 regeneration cycles without loss of binding capacity [308], [309]. Recently, Nb-based matrices specific for GFP/YFP and mCherry fluorescent proteins have been developed for the purification of fusion proteins, such as recombinant human topoisomerase IIα, achieving yields up to 5.2 mg/L from HEK293F cultures [310]. A refined approach employs lentiviral transduction to express GFP- or ALFA (15-amino acid peptide tag)-tagged target proteins; Nb columns then capture and purify these fusions under native conditions. Cleavage using engineered SUMO protease SENP^EuB^ at 4 ℃ enables gentle elution of soluble proteins even from challenging targets [311]. This workflow provides a universal, regenerable, and cost-efficient affinity platform for proteomic research.

#### 5.3.3 Facilitating target protein degradation

Direct degradation of proteins at the post-translational level offers an efficient route for functional knockdown compared to genetic or RNA-based methods [312]. Fusing Nbs to the really interesting new gene (RING) domain of the E3 ubiquitin ligase RING finger protein 4 (RNF4) has enabled rapid proteasomal degradation of Nb-tagged targets within minutes following intracellular delivery [313]. Additionally, more advanced systems, such as multivalent Nb-targeted chimeras (mNbTACs), exploit endocytic pathways to drive lysosomal degradation. For example, ^Dox^o-mvNbs^PPH^ carrying doxorubicin specifically targets PD-L1 and HER2, promoting their lysosomal sequestration and triggering immunogenic cell death with potent antitumor effects [314]. Nb-mediated degradation has also been extended to extracellular and membrane proteins. A proteolysis-targeting chimera (PROTAC)-like strategy called GlueTAC couples Nbs to cell-penetrating peptides and lysosomal sorting sequences, thereby enabling internalization and degradation of otherwise “undruggable” surface molecules such as PD-L1 [315], [316].

#### 5.3.4 Assisting protein structure determination

Obtaining high-resolution structural information for dynamic or membrane proteins remains challenging [317]. Nbs, acting as crystallization chaperones, stabilize specific conformational states and facilitate crystallization or cryo-electron microscopy (cryo-EM) analysis [318]. For G protein-coupled receptors (GPCRs) with small size and membrane-embedded characteristics, such as neurotensin receptor 1 and the μ-opioid receptor, Nbs recognizing intracellular loops have enabled visualization of inactive-state structures at near-atomic resolutions comparable to those achievable by X-ray crystallography [319]. In addition, attaching Nbs to large scaffolds can mitigate preferred-orientation artifacts in cryo-EM, improving particle alignment and resolution [320]. Extending this principle, the NabFab platform integrates Nbs with Fab-scaffold adaptors, substantially enhancing cryo-EM imaging efficiency. NabFab has enabled high-resolution characterization of membrane transporters, including vcNorM and *Streptomyces* capitis divalent metal transporter (ScaDMT) [321].

#### 5.3.5 Intracellular protein research and regulation

Nbs can be expressed stably within cells, known as intrabodies, allowing real-time monitoring and manipulation of intracellular targets [322], [323]. GFP-binding Nbs have been employed to construct “nanotraps” that recruit GFP-tagged proteins to specific cellular compartments and modulate their activity in live cells [63]. With further research, co-expression systems combining intracellular Nbs with fluorescent reporters facilitate endogenous protein tracking and high-content analysis (HCA) [324]. Beyond visualization, Nb fusions have enabled manipulation of post-translational modifications. For example, linking a depalmitoylase to a β-subunit-specific Nb reduced Ca(v)1.2 palmitoylation, altering channel voltage dependence and arrhythmogenic risk in cardiomyocytes [325]. Nbs also provide analytical utility for protein-protein interaction (PPI) mapping. A NanoLuc luciferase fragment complementation assay uses two primary Abs to bind two targets and secondary Nbs fused to split luciferase fragments. Once the two target proteins interact, this proximity relationship enables these two luciferase fragments to reconstitute active NanoLuc, yielding a luminescence signal that quantitatively visualizes endogenous PPIs *in situ* [326].

#### 5.3.6 Emerging nanobody-based technologies

Recent methodological innovations have expanded Nb utility in molecular biology and biotechnology. Nb-tethered transposition sequencing (NTT-seq) integrates recombinant Tn5 transposase with distinct secondary Nbs (nb-Tn5), enabling simultaneous detection of multiple histone modifications and DNA-binding proteins at single-cell resolution. NTT-seq can capture up to three epigenetic features genome-wide in a single experiment that generates multimodal chromatin landscape maps [327]. Programmable antigen-gated engineered GPCRs (PAGERs) exemplify another emerging class. These synthetic receptors incorporate a conditionally autoinhibitory Nb domain into GPCR scaffolds, allowing antigen binding to relieve inhibition and initiate downstream signaling. PAGERs can be tuned for antigen-regulated transgene expression, fluorescent signaling, or G-protein activation, thus offering a flexible tool for cellular engineering and drug discovery [328]. In gene-delivery research, Nb-adeno-associated virus (AAV) conjugates (NACs) have been created by covalently coupling targeting Nbs to AAV capsids through valine-guanine linker chemistry. NACs enable customizable tropism and improved cell-specific gene delivery, expanding recombinant AAV (rAAV) applications in targeted imaging and therapeutic intervention [329].

### 5.4 Challenges faced in the application of nanobodies

Despite the rapid progress of Nbs in research and diverse biomedical applications, several critical challenges remain unresolved. These include potential immunogenicity, off-target or systemic effects, and difficulties in large-scale manufacturing and cost-efficient production. Although the immunogenicity of Nbs is markedly lower than that of conventional monoclonal Abs, certain structural features outside the human Ab repertoire, such as exposed VH-VL interface residues or aggregation-prone regions, may confer intrinsic immunogenic potential [6], [330]. It is not uncommon for candidates exhibiting unexpected immune responses to appear during preclinical development [331], [332], [333]. Nevertheless, careful optimization of drug formulation and administration routes can mitigate such risks, allowing some molecules to proceed successfully to market authorization [197]. Comprehensive assessment of immunogenic epitopes, structural aggregation, and formulation stability, therefore, remains essential in clinical translation. As for the off-target effects or adverse reactions, engineering approaches that enhance tissue specificity, such as targeted conjugation or modular bispecific design, represent effective strategies to alleviate these concerns [334]. At last, the large-scale production of Nbs constitutes another major challenge [335]. Conventional *E. coli* systems often yield endotoxin-contaminated preparations due to the presence of LPS. Therefore, under most current good manufacturing practice (cGMP)-compliant processes, Nb production is typically carried out using CHO cells and *P. pastoris* expression systems [336], [337]. Recent work using the Gram-negative bacterium *Acinetobacter baumannii*, which naturally lacks LPS, achieved high-level extracellular secretion of VHHs through the Omp38 signal peptide, reducing endotoxin levels by approximately 2×10⁵-fold [338]. This innovation provides a promising avenue for scalable, low-cost, and high-purity Nb manufacturing.

## 6 Future perspectives of nanobodies

### 6.1 Integration with artificial intelligence (AI) and computational design

Recent advances in AI have profoundly accelerated the discovery, design, and optimization of Nbs [339], [340]. Efficient engineering requires accurate prediction of protein tertiary structures, and state-of-the-art deep-learning algorithms such as AlphaFold 3 [341] and RoseTTAFold [342] have achieved unprecedented success in predicting Ab-antigen interactions and folding architectures. These tools enable the rapid identification of high-quality candidate Nbs, greatly reducing reliance on empirical screening.

Currently, the trend of de novo protein design is reshaping computational biology [343], [344], [345], [346]. With algorithms including RFDiffusion [347], [348], [349], ProteinMPNN [350], AlphaProteo [351], BindCraft [352], ODesign [353], and Boltzdesign1 [354], researchers can now design high-affinity binders in silico. Concurrently, a series of complementary algorithmic innovations, including Chai-2 [355], IgGM [356], BoltzGen [357], JAM [358], mBER [359], Germinal [360], AntiFold [361], and AntiBMPNN [362], have been applied to de novo design of Nbs and demonstrated a relatively high success rate. Currently, Zhao et al. [363] engineered Nbs against proliferating cell nuclear antigen (PCNA) and B-cell lymphoma 6 protein (BCL6) by redesigning the CDR3 loops and grafting them onto optimized scaffolds. A study conducted by the Baker laboratory further demonstrated that fine-tuned AI models can reach atomic-level precision when designing VHHs for defined antigens [364]. Beyond structural prediction and design, AI is being leveraged to construct Nb libraries using large protein language models [365] or to forecast mutation effects and binding affinity, rapidly identifying promising variants for experimental validation [366]. These developments underscore a future in which AI-assisted Nb design becomes a central paradigm of next-generation therapeutics [367].

Potential immunogenicity remains a key obstacle in clinical translation. By analyzing sequence features and immune epitope profiles, AI models can forecast immunogenic risk and toxic liabilities of candidate Nbs [368], [369]. Moreover, AI-guided thermal stability predictions can substantially reduce the cost of experimental screening for engineered variants [370], [371]. Nevertheless, current computational tools still show limited reliability in accurately modeling the diverse CDR3 conformations that dominate Nb specificity. It was reported that even for the relatively superior AlphaFold 3 model, the high accuracy success rate in predicting the docking of Nbs with antigens was only 13.3% [372], [373], [374]. Therefore, theoretical predictions must be complemented by rigorous experimental validation. As structural databases expand and machine-learning frameworks mature, the predictive accuracy, particularly for antigen-binding interfaces and paratope conformers, is expected to significantly improve.

### 6.2 Convergence with emerging therapeutic modalities

**Fig. 7:**
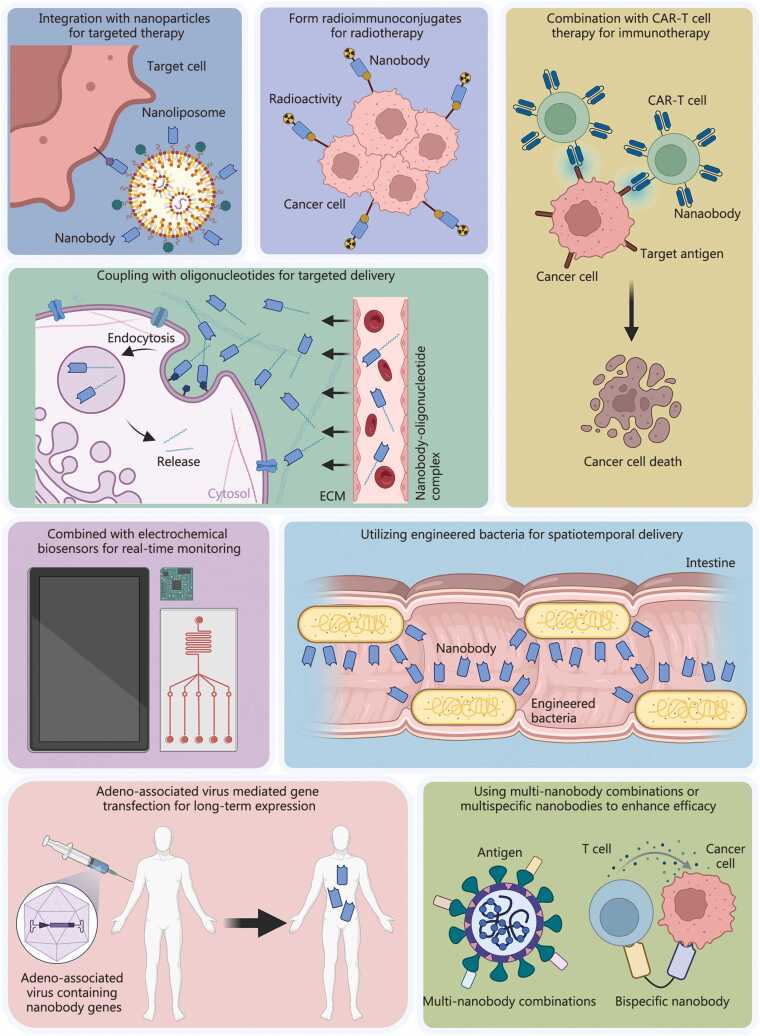
Innovative therapeutic strategies integrating nanobodies (Nbs) in precision medicine. CAR-T. Chimeric antigen receptor T-cell; ECM. Extracellular matrix.

Although several Nb-based biologics have already been approved for clinical use **(**Table 2**)**, Nb-based technology remains an evolving frontier undergoing continuous refinement. Multimodal combinations and hybrid platforms are expanding therapeutic horizons: 1) Nb-nanoparticle conjugates for targeted drug delivery [375]; 2) Nb-CAR-T constructs for enhanced immunotherapy [376]; 3) radiolabeled Nbs forming radioimmunoconjugates for precision radiotherapy [377]; 4) Nb-oligonucleotide conjugates improving nucleic acid delivery and diagnostics [378]; 5) electrochemical biosensors integrating Nbs for real-time molecular monitoring [379]; 6) engineered probiotic strains for spatially controlled Nb release *in situ* [181], [380]; 7) AAV-mediated long-term expression of Nbs *in vivo* [381]; 8) multivalent or multispecific Nb cocktails for synergistic efficacy and reduced systemic toxicity [155], [382], [383]. Together, these innovations position Nbs as a cornerstone of precision and personalized medicine, bridging immunotherapy, gene delivery, imaging, and molecular diagnostics **(**Fig. 7**)**.

## 7 Conclusions

As unique single-domain antigen affinity fragments, Nbs have become a transformative tool in biomedicine owing to compact size, high stability, superior solubility, and exceptional target specificity. This review has systematically highlighted recent advances encompassing their molecular structure, generation and optimization, production technologies, and multifaceted applications across therapeutics, diagnostics, and research. Nbs have displayed effectiveness against tumors, toxins, infectious and cardiovascular diseases, and have contributed invaluable utilities as molecular probes, imaging agents, and biotechnological tools. Nevertheless, for successful clinical translation, key challenges must still be addressed, namely, biological safety, immunogenicity control, and scalable cost-efficient manufacturing. Looking forward, the integration of AI-driven design, molecular engineering, and multidisciplinary platforms is expected to unleash the full translational capability of Nb-based interventions. As these trends converge, Nbs are poised to become a central driving force in next-generation precision medicine, drug discovery, and biotechnology innovation.

## Declarations

None

## Ethics approval and consent to participate

Not applicable.

## Funding

This work was supported by the Zhejiang Provincial Top Discipline Program (School of Pharmaceutical Sciences, Wenzhou Medical University), Wenzhou Medical University Talent Research Start-up Project (89225016), and the Open Fund of Key Laboratory of Structural Malformations in Children of Zhejiang Province (ZJET2302Z).

## CRediT authorship contribution statement

XKL and ZJS conceived and designed the study. CKC, XYX, and FL contributed to the data acquisition. CKC prepared figures and tables. MY and YYC helped revise the manuscript. CKC and ZJS draft the manuscript. All authors read and approved the final manuscript.

## Declaration of Competing Interest

The authors declare that they have no competing interests.

## Data Availability

Not applicable.
